# Nickel Phosphine Complexes: Synthesis, Characterization, and Behavior in the Polymerization of 1,3-Butadiene

**DOI:** 10.3390/molecules30234655

**Published:** 2025-12-04

**Authors:** Massimo Guelfi, Giulio Bresciani, Guido Pampaloni, Anna Sommazzi, Francesco Masi, Benedetta Palucci, Simona Losio, Giovanni Ricci

**Affiliations:** 1 Dipartimento di Chimica e Chimica Industriale, Università di Pisa, Via Moruzzi 13, I-56124 Pisa, Italy; giulio.bresciani@unipi.it; 2 Centro per l’Integrazione della Strumentazione Scientifica dell’Università di Pisa (C.I.S.U.P), Università di Pisa, I-56124 Pisa, Italy; 3 Independent Researcher, I-56025 Pontedera, Italy; pampaloniguido55@gmail.com; 4 Independent Researcher, I-28100 Novara, Italy; anna.sommazzi14@gmail.com; 5 Independent Researcher, I-26866 Sant’Angelo Lodigiano, Italy; rolando.masi54@gmail.com; 6 CNR—Istituto di Scienze e Tecnologie Chimiche “Giulio Natta” (SCITEC), Via A. Corti 12, I-20133 Milano, Italy; benedetta.palucci@scitec.cnr.it (B.P.); simona.losio@scitec.cnr.it (S.L.)

**Keywords:** nickel, phosphine complexes, crystalline structures, polymerization, polybutadiene

## Abstract

Several nickel dichloride phosphine complexes have been synthesized, their crystalline structure determined, and their behavior, in combination with methylaluminoxane, in the polymerization of butadiene has been examined. High-*cis* polybutadienes were consistently obtained, regardless of the nature of the phosphine coordinated to the metal and the methylaluminoxane/Ni molar ratio used, contrary to what was previously observed in the polymerization of butadiene with analogous cobalt phosphine complexes, in which catalytic selectivity was found to be strongly influenced by these two factors. An interpretation for such different behavior is provided.

## 1. Introduction

Nickel- and cobalt-based catalysts are well known in the field of polymerization of butadiene and other substituted butadienes and are also characterized by very similar behavior [1]. For example, the Co(acac)_2_-AlEt_2_Cl-H_2_O [2] and AlEt_3_-Ni(carboxylate)-BF_3_·OEt_2_ [3,4] systems are of industrial interest for the production of *cis*-1,4 polybutadiene; systems obtained by combining Ni(acac)_2_ and Co(acac)_2_ with MAO provide highly *cis*-1,4 polymers from butadiene and syndiotactic *cis*-1,4 polymers from 1,3-pentadiene and 3-methyl-1,3-pentadiene [5,6,7]. Likewise, various catalytic systems based on nickel and cobalt complexes with various types of ligands containing nitrogen and oxygen atoms as donor atoms, in combination with MAO, provide polybutadiene with a high content of *cis*-1,4 units [8,9,10].

Previous work on the polymerization of butadiene with catalysts based on cobalt phosphine complexes has highlighted how catalytic selectivity is strongly affected by the nature of the phosphine ligand coordinated to the metal and by the MAO/Co molar ratio [11,12,13,14,15,16,17,18]. Given the similarity between cobalt and nickel, we sought to verify whether analogous results could be achieved with nickel phosphine complexes. To this end, we synthesized several nickel phosphine complexes, determined their crystalline structures, and studied their behavior in the polymerization of butadiene.

The results obtained are reported in this paper, along with a plausible interpretation.

## 2. Results

### 2.1. Synthesis of Nickel Complexes

**Scheme 1:**
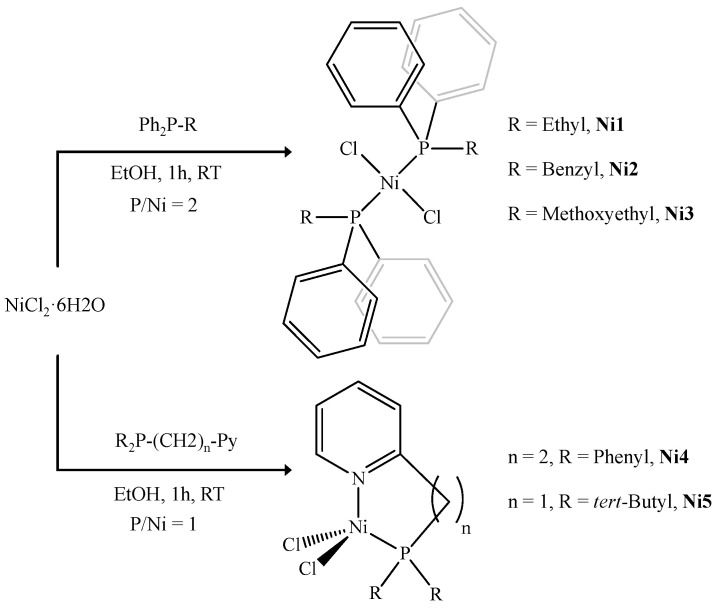
Synthesis conditions for **Ni1**–**Ni5** complexes.

To investigate the catalytic potential of phosphine complexes in diene polymerization reactions, two types of ligands were selected to obtain complexes with distinct structural features and varying degrees of steric bulk around the metal center: monodentate phosphines (Ph_2_PR) and chelating pyridyl-phosphines (R_2_P–(CH_2_)_n_–Py). Complexes of the general type, NiX_2_L_2_ (X = halide; L = tertiary phosphine or amine), have been known of for decades, and both tetrahedral (paramagnetic) and square-planar (diamagnetic) forms are well documented in early foundational studies [19,20,21,22,23,24]. The reaction of NiCl_2_·6H_2_O with two equivalents of Ph_2_PR in ethanol at room temperature yielded neutral, [NiCl_2_(Ph_2_PR)_2_] complexes (see Scheme 1) in high yields. Furthermore, compound **Ni1** can also be synthesized using NiCl_2_ in toluene at 90 °C for 4 h (see Materials and Method section). Reacting NiCl_2_·6H_2_O with one equivalent of R_2_P–(CH_2_)_n_–Py under the same conditions as those described above led to the formation of compounds in which the phosphine acts as a chelating ligand (Scheme 1).

The attempts to isolate bis-substituted products using two equivalents of the ligand were unsuccessful. Complex **Ni4** has already been reported in the literature [25,26] using slightly different conditions. Complexes **Ni1**–**Ni5** were characterized by elemental analysis, FT-IR (Figures S1–S5) and SCXRD (in the case of **Ni1**, **Ni3,** and **Ni5**). All isolated complexes displayed colors ranging from dark red to violet, except for **Ni4**, which appeared green in the solid state (Figure S6). In solution, however, **Ni4** exhibits a red-violet color (Figure S7). This behavior is fully consistent with previous reports [25,26], where the green, solid-state form was identified as a dimeric species with bridging chloride atoms, while the red-violet solution form corresponds to the monomeric analog. All square-planar complexes (**Ni1**–**Ni3**) were found to be diamagnetic, consistent with low-spin configurations in the solid state. However, in chloroform solution, they exhibited a reasonably fluxional behavior between square-planar and tetrahedral geometries, as indicated by the effective magnetic moments, which ranged from 1.97 to 2.09 μ_B_ [27,28].

### 2.2. Crystallographic Characterization

**Figure 1:**
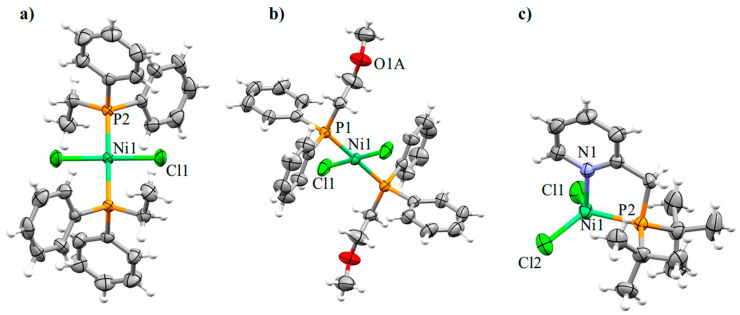
Molecular structures of complexes (**a**) **Ni1**; (**b**) **Ni3**; and (**c**) **Ni5**. Ellipsoids are drawn at the 50% probability level. Nickel atoms are shown in bluish green, chlorine in green, phosphorus in orange, nitrogen in light blue, and oxygen in red. Atom numbering is consistent with that reported in the CIF file; for clarity, symmetry-equivalent atom labels have been omitted.

Complexes **Ni1** and **Ni3** exhibit an almost perfectly square-planar geometry (Figure 1), as indicated by Cl–Ni–P bond angles of 90.52° (Cl1–Ni–P2) and 91.30° (Cl1–Ni–P1), measured within the asymmetric units of **Ni1** and **Ni3**, respectively (Table S1). The Ni–P bond lengths are in excellent agreement with values typically observed in related phosphine–nickel complexes [29] (Figure S8).

In contrast, complex **Ni5** adopts a pseudo-tetrahedral geometry (Figure 1), with a τ_4_′ value of 0.86 [30]. However, its geometry can be more accurately described as a distorted trigonal pyramid, based on the low value of δ = 8.994, compared to the ideal tetrahedral reference (δ = 15.765), both computed using the *Polynator 1.7.1* software [31]. Atomic deviations from ideal positions fall within the 20.0–41.4 pm range (Figure S9). The Ni–P bond lengths in **Ni5** are slightly longer than those in **Ni1** and **Ni3**, consistent with the structural rigidity imposed by the chelating ligand (Table S1). Within the lattice, a series of weak contacts are established involving the chlorine, oxygen, and hydrogen atoms of the phosphine ligands (Table S2 and Figures S10–S13).

**Figure 2:**
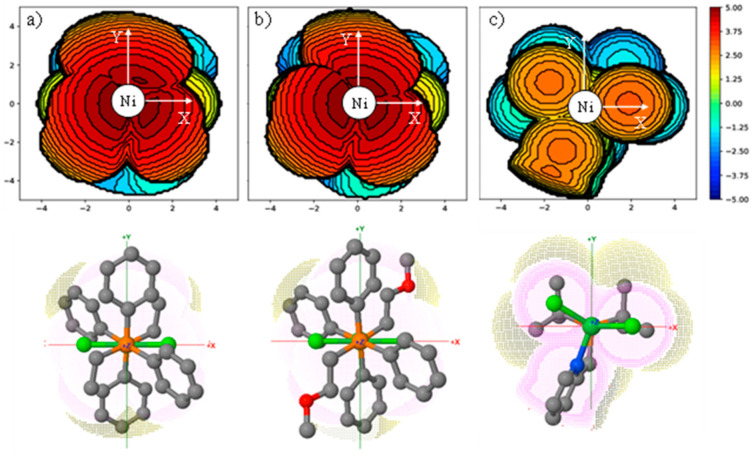
Topographic steric maps of the phosphine ligands in complexes (**a**) **Ni1**, (**b**) **Ni3**, and (**c**) **Ni5**. The nickel atom is placed at the center of a sphere with a radius of 5 Å, the Z-axis is defined along the Ni–P bond vector, and the projections are displayed in the XY plane. The default Bondi radii, scaled by 1.17, was left unchanged, as was the mesh spacing for numerical integration, which was set to 0.10.

To assess the steric hindrance of the phosphine ligands, the percentage buried volume (%V_bur_) was calculated from the crystallographic data using the *SambVca 2.1* web tool [32,33,34] (Figure 2). Complexes **Ni1** and **Ni3** exhibit comparable steric profiles, with %V_bur_ values of 64.7% and 64.0%, respectively. In contrast, complex **Ni5** features a significantly lower buried volume (%V_bur_ = 52.0%), in line with the presence of a less sterically demanding phosphine (Table S3).

### 2.3. Polymerization of 1,3-Butadiene

The results obtained in the polymerization of 1,3-butadiene with the catalysts obtained by combining the nickel phosphine complexes with MAO are shown in Table 1 and can be summarized as follows.

**Figure 3:**
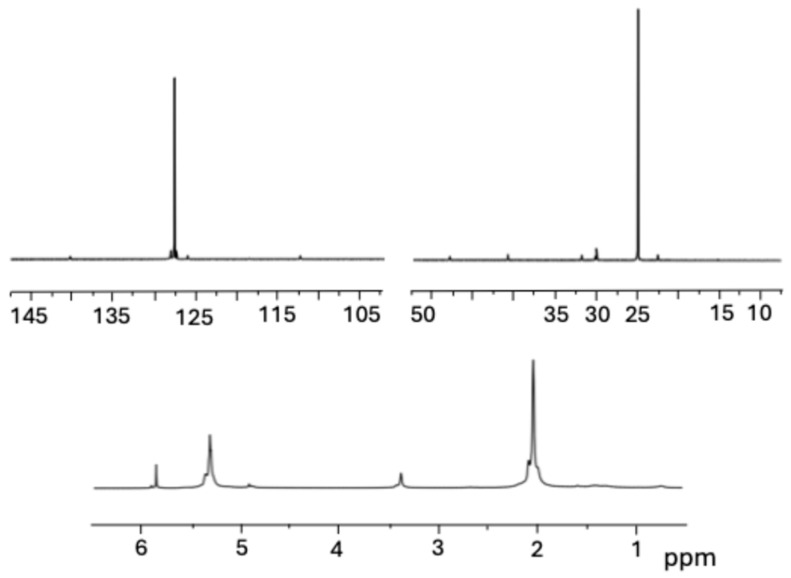
^13^C (**up**) and ^1^H (**down**) NMR of polybutadiene in Table 1, entry 2 (C_2_D_2_Cl_4_ as solvent, HMDS as internal standard, 103 °C).

All the catalytic systems used yield polybutadiene with an essentially *cis* structure (>92%), as is evident from the NMR (^1^H and ^13^C) (Figure 3 and Figure S14–S16) and FT-IR (Figure S17) spectra of the polymers themselves, regardless of the type of catalyst, i.e., the nature of the phosphine ligand and the MAO/Ni molar ratio.

The slight variations observed in the *cis* content of the polymers are insufficient to establish a correlation between the catalytic selectivity and the nature of the phosphine ligand or the MAO/Co molar ratio, as is possible in the case of similar cobalt-based catalytic systems.

The polymers obtained exhibit a rather low molecular weight (in the range 30,000–56,000 g·mol^−1^) and a narrow molecular weight distribution. The polymerization rate is not particularly high, even if complete monomer conversions are achieved within a few hours.

## 3. Discussion

As mentioned above, in the case of the polymerization of butadiene with completely analogous cobalt-based catalyst systems, a significant influence of the type of ligand and the MAO/Co molar ratio on the catalytic selectivity, but also on the catalytic activity, was observed [11,12,13,14,15,16,17,18].

Depending on the type of phosphine coordinated to the metal (i.e., aliphatic, aromatic, or bidentate also containing a nitrogen atom as a donor atom) and the MAO/Co molar ratio, it was possible to obtain polybutadienes with essentially *cis* structures, mixed *cis*-1,4/1,2 structures, 1,2-, iso-, or syndiotactic structures.

**Figure 4:**
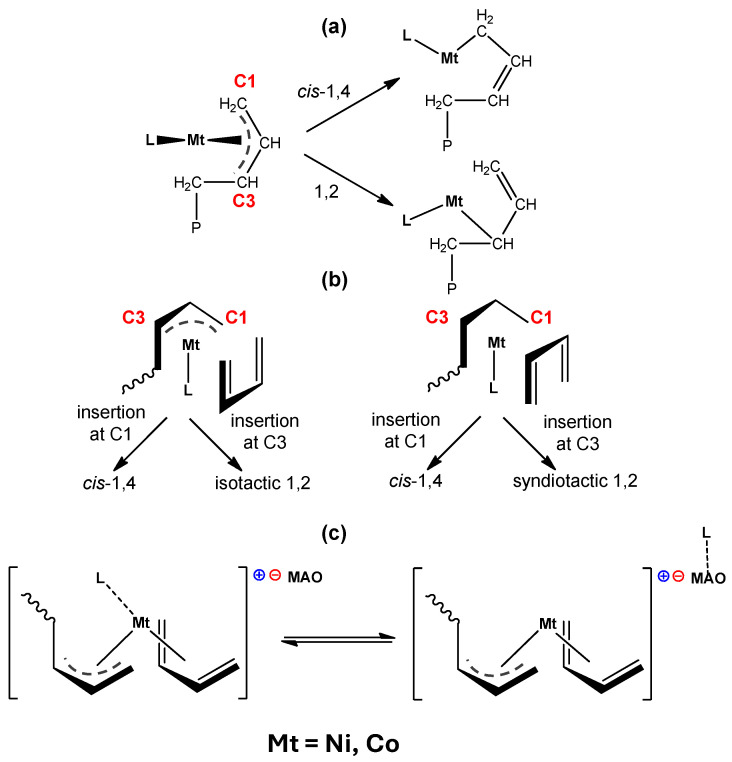
How the phosphine ligand can affect the formation of *cis*-1,4, and 1,2, iso- and syndiotactic polybutadienes: (**a**) by favoring the insertion of the incoming monomer at C1 or C3 of the butenyl group; (**b**) by influencing the mutual orientation (*exo-exo* or *exo-endo*) of incoming monomer and butenyl group; (**c**) by migrating from the metal to methylaluminoxane and thus causing a change in selectivity.

Plausible mechanisms were hypothesized to explain the formation of *cis*-1,4, and 1,2, iso- and syndiotactic polymeric structures [35]. The phosphine ligand, depending on its steric hindrance, can influence the entrance of the new monomer onto the allylic unit by favoring the incoming at C1 or C3, with the formation of a 1,4 or 1,2 unit (Figure 4a); the phosphine ligand is also able to influence the mutual orientation of the incoming butadiene and of the allylic unit, thus favoring, through the insertion of the incoming butadiene at C3 of the allylic unit, the formation of an iso- or syndiotactic 1,2 polymer (Figure 4b). Furthermore, depending on the type of ligand and the value of the MAO/Co molar ratio, the ligand can be abstracted from the metal, leading to the formation of a catalytic center such as that deriving from the simple combination of MtCl_2_ and MAO, specifically forming an exclusively *cis*-1,4 polymer (Figure 4c).

The latter (Figure 4c) is likely what happens in the case of nickel-based systems, since they invariably yield a polymer with an essentially *cis* structure regardless of the catalytic structure: the ligand is transferred to methylaluminoxane, generating the active species responsible for *cis* polymer formation.

## 4. Materials and Methods

### 4.1. General Procedures and Materials

For the synthesis of the complexes, reactants and solvents were purchased from Merck KGaA (Darmstadt, Germany) or Strem Chemicals (Ascensus Specialties, Bellevue, WA, USA) and were of the highest purity available. Complexes **Ni2** [36] and **Ni4** [25,26] were synthesized with a slightly modified version of the procedure described in the literature. Reactions were conducted under N_2_ atmospheric conditions using standard Schlenk techniques, and all products were stored in air once isolated. Solvents were used as received unless otherwise stated. Toluene and diethyl ether were dried with the solvent purification system mBraun (Garching, Germany) MB SPS5. IR spectra of solid samples were recorded on Agilent (Santa Clara, CA, USA) Cary630 FTIR spectrometer. Elemental analyses were performed on a Vario MICRO cube instrument (Elementar, Cheadle, UK).

For the synthesis and characterization of the polybutadienes, methylaluminoxane (MAO) (Merck, 10 wt% solution in toluene) and deuterated solvent for NMR measurements (C_2_D_2_Cl_4_) (Merck, >99.5% atom D) were used as received. Toluene (Merck, 99.8% pure) was refluxed over Na for *ca*. 8 h, then distilled and stored over a molecular sieve under dry dinitrogen. Prior to each run, 1,3-butadiene (Merck, ≥99%) was evaporated from the container, dried by passing it through a column packed with molecular sieves, and condensed into the reactor which had been precooled to −20 °C.

### 4.2. Synthesis of [NiCl_2_(ethyldiphenylphosphine)_2_] (***Ni1***)

(a) To a solution of NiCl_2_·6H_2_O (278 mg, 1.16 mmol) in degassed ethanol (20 mL), ethyldiphenylphosphine (500 µL, 2.32 mmol) was added. The resulting suspension was stirred at room temperature for 1 h. The suspension was then filtered, and the resulting red solid was washed with diethyl ether (2 × 10 mL) and dried under vacuum at 40 °C, affording 599 mg (92% yield) of a red solid corresponding to C_28_H_30_Cl_2_NiP.

(b) To a suspension of NiCl_2_ (150 mg, 1.16 mmol) in toluene (10 mL), ethyldiphenylphosphine (500 µL, 2.32 mmol) was added. The mixture was stirred at 90 °C for 4 h. The solvent was removed under reduced pressure, and the resulting red solid was washed with diethyl ether and dried under vacuum at 40 °C, yielding 534 mg (82% yield) of a red solid identical to that obtained in method (a). Single crystals suitable for SCXRD analysis were grown by slow vapor diffusion of hexane into a dichloromethane solution of the complex. Elemental analysis (%): Calcd. for C_28_H_30_Cl_2_NiP: C, 58.35; H, 4.08. Found: C, 58.23; H, 4.43. Selected IR data (solid state, cm^−1^): 1432 (m), 1101 (m), 1000 (m), 757 (m), 737 (s), 705 (s), and 692 (s) (Figure S1).

### 4.3. Synthesis of [NiCl_2_(benzyldiphenylphosphine)_2_] (***Ni2***)

To a solution of NiCl_2_·6H_2_O (108 mg, 0.45 mmol) in degassed ethanol (10 mL), benzyldiphenylphosphine (250 mg, 0.91 mmol) was added. The resulting suspension was stirred at room temperature for 1 h. The mixture was filtered, and the resulting red solid was washed with diethyl ether (2 × 5 mL) and dried under vacuum at 40 °C, affording 188 mg (x% yield) of a red solid with composition C_38_H_34_Cl_2_NiP. Elemental analysis (%): Calcd. for C_38_H_34_Cl_2_NiP: C, 70.08; H, 5.26. Found: C, 69.83; H, 5.39. Selected IR data (solid state, cm^−1^): 1434 (m), 1000 (m), 836 (s), 776 (m), 740 (s), and 690 (s) (Figure S2).

### 4.4. Synthesis of [NiCl_2_(2-methoxyethyldiphenylphosphine)_2_] (***Ni3***)

To a solution of NiCl_2_·6H_2_O (198 mg, 0.83 mmol) in degassed ethanol (20 mL), 2-methoxyethyldiphenylphosphine (405 mg, 1.66 mmol) was added. The resulting suspension was stirred at room temperature for 1 h. The solid was collected by filtration, washed with diethyl ether (2 × 5 mL), and dried under vacuum at 40 °C, affording 391 mg (76% yield) of a red solid corresponding to C_30_H_34_Cl_2_NiO_2_P_2_. Single crystals suitable for SCXRD analysis were grown via the slow vapor diffusion of hexane into a dichloromethane solution of the complex. Elemental analysis (%): Calcd. for C_30_H_34_Cl_2_NiO_2_P_2_: C, 58.29; H, 5.54. Found: C, 58.13; H, 5.62. Selected IR data (solid state, cm^−1^): 1484 (w), 1439 (m), 1186 (w), 1000 (s), 978 (m), 953 (m), 745 (s), and 700 (s) (Figure S3).

### 4.5. Synthesis of [NiCl_2_(k2-N,P-2-(2-(diphenylphosphino)ethyl)pyridine)] (***Ni4***)

To a solution of NiCl_2_·6H_2_O (204 mg, 0.86 mmol) in degassed ethanol (10 mL), 2-(2-(diphenylphosphino)ethyl)pyridine (250 mg, 0.86 mmol) was added. The mixture was stirred at room temperature for 1 h. The solvent was removed under reduced pressure; the resulting red solid was washed with diethyl ether and dried under vacuum to afford 319 mg (88% yield) of a green solid corresponding to C_19_H_18_Cl_2_NNiP. Elemental analysis (%): Calcd. for C_19_H_18_Cl_2_NNiP: C, 54.21; H, 4.31. Found: C, 53.99; H, 4.45. Selected IR data (solid state, cm^−1^): 1606 (m), 1485 (m), 1439(m), 1432 (m), 1160 (w), 1101 (w), 757 (s), 744 (s), 718 (s), and 698 (s) (Figure S4).

### 4.6. Synthesis of [NiCl_2_(k2-N,P-2-((di-tert-butylphosphino)methyl)pyridine)] (***Ni5***)

To a solution of NiCl_2_·6H_2_O (200 mg, 0.84 mmol) in degassed ethanol (10 mL), 2-((di-tert-butylphosphino)methyl)pyridine (200 mg, 0.84 mmol) was added. The reaction mixture was stirred at room temperature for 1 h. The solvent was removed under reduced pressure; the resulting purple solid was washed with diethyl ether and dried under vacuum, affording 290 mg (94% yield) of a purple solid. Single crystals suitable for SCXRD analysis were grown via the slow vapor diffusion of hexane into a dichloromethane solution of the complex. Elemental analysis (%): Calcd. for C_14_H_24_Cl_2_NiP: C, 45.83; H, 6.59. Found: C, 45.66; H, 6.69. Selected IR data (solid state, cm^−1^): 1606 (m), 1485 (m), 1439(m), 1181 (w), 826 (s), 771 (s), and 761 (Figure S5).

### 4.7. SCXRD

Single-crystal X-ray diffraction (SCXRD) was performed using a Bruker D8 Venture instrument equipped with a microfocus Mo source (Kα radiation, λ = 0.71073 Å) and a 2D Photon III detector. The main experimental details regarding the determination of the structure of **Ni1**, **Ni3,** and **Ni5** by SCXRD are reported in Table S4. The unit cells were identified and initially refined using APEX4 [37]. Successively, data were integrated and reduced using SAINT [38] and XPREP [39]. Absorption effects were corrected using SADABS [40]. Structure was solved and refined with the aid of SHELXL-2019/1 [41]. The oxygen atom O1 in **Ni3** and the carbon atom C8 in **Ni5** exhibited irregular anisotropic parameters due to disorder, and their electron density was modeled at multiple positions. The occupancies were freely refined to convergence using the FVAR command. All hydrogen atoms were fixed at calculated position and refined using a riding model. Crystallographic data of **Ni1**, **Ni3,** and **Ni5** can be obtained free of charge from the Cambridge Crystallographic Data Center (CCDC number: 2496672 for **Ni1**, 2496673 for **Ni3** and 2496674 for **Ni5**). The molecular structures of all complexes are depicted in Figure 1 and selected bond lengths and angles are listed in Table S4.

### 4.8. Polymerization of 1,3-Butadiene

Polymerizations were carried out in a 25 mL round-bottomed Schlenk flask. A standard procedure is reported. Prior to starting polymerization, the reactor was heated to 110 °C under vacuum for 1 h and backfilled with nitrogen. 1,3-Butadiene was condensed into the Schlenk flask kept at −20 °C, then toluene was added, and the solution was brought to the desired polymerization temperature. MAO and a toluene solution of the nickel complex were then added in that order. Polymerization was stopped with methanol containing a small amount of hydrochloric acid. The polymer obtained was then coagulated by adding 40 mL of a methanol solution containing 4% of Irganox^®^ 1076 antioxidant and HCl, and repeatedly washed with fresh methanol, and finally dried in vacuum at room temperature to constant weight.

### 4.9. Polymer Characterization

Attenuated total reflectance (ATR)–Fourier transform infrared spectroscopy (FTIR) spectra were recorded at room temperature in the 4000–600 cm^−1^ range with a resolution of 4 cm^−1^ using a Perkin Elmer (Waltham, MA, USA) Spectrum Two spectrometer. NMR spectra were recorded on a Bruker (Billerica, MA, USA) Advance 400 MHz NMR Spectrometer operating at 400 MHz (^1^H) and 100.58 MHz (^13^C) working in the PFT mode at 103 °C. NMR samples were prepared dissolving from 60 to 80 mg of polymer in about 3 mL of C_2_D_2_Cl_4_ in 10mm probes and referred to as hexamethyldisiloxane (HMDS), as the internal standard. The relaxation delay was 16 s. The microstructure of the polymers was determined by ^1^H and ^13^C NMR, according to the literature [42,43].

The molecular weight average (*M*_w_) and the molecular weight distribution (*M*_w_/*M*_n_) were obtained by a high-temperature Waters (Milford, MA, USA) GPCV2000 size exclusion chromatography (SEC) system equipped with a refractometer detector. The experimental conditions consisted of three PL Gel Olexis columns, *ortho*-dichlorobenzene (DCB) as the mobile phase, a 0.8 mLmin^−1^ flow rate, and a temperature of 145 °C. The calibration of the SEC system was constructed using eighteen narrow *M*_w_/*M*_n_ PS standards with molar weights ranging from 162 to 5.6·10^6^ g mol^−1^. For the SEC analysis, about 12 mg of polymer was dissolved in 5 mL of DCB with 0.05% of BHT as antioxidant.

## 5. Conclusions

We synthesized and characterized several nickel dichloride complexes with various types of phosphine ligands. Given the similar behavior exhibited by nickel- and cobalt-based catalytic systems in the polymerization of butadiene, we wanted to verify whether, in the case of nickel, as happened in the case of cobalt, it was possible to control and vary the catalytic selectivity, i.e., the formation of polymers with *cis*-1,4, and 1,2, iso- and syn-diotctic structures, by varying the type of phosphine ligand on the nickel atom. However, this was not the case, as polybutadienes with a high *cis* content were consistently obtained, regardless of the nature of the phosphine ligand.

This result was interpreted by assuming that the ligand is removed from the metal, which results in the formation of an active center completely analogous to that obtained by simply combining NiCl_2_ or Ni(acac)_2_ with MAO, which, as it is well known, is specific to produce *cis* polybutadiene.

## Figures and Tables

**Table 1:** Polymerization of 1,3-butadiene with nickel catalysts ^a^.

Entry	Ni_Complex	MAO/Ni (Molar Ratio)	Time (h)	Yield (g)	N ^b^ (h^−1^)	*cis*-1,4 ^c^ (%)	M_w_ ^d^ (g/mol)	M_w_/M_n_ ^d^
entry 1	Ni1	100	3	1.4	864	92.3	41,000	2.5
entry 2	Ni1	1000	3	1.4	864	94.2	44,000	1.5
entry 3	Ni2	100	3	1.4	864	92.0	32,000	3.0
entry 4	Ni2	1000	3	1.4	864	94.2	56,000	2.1
entry 5	Ni3	100	4	1.19	550	95.3	46,000	3.1
entry 6	Ni3	1000	4	1.4	648	97.1	48,500	2.8
entry 7	Ni4	100	3	1.12	691	96.0	30,000	2.4
entry 8	Ni4	1000	3	1.30	802	96.4	35,000	2.2
entry 9	Ni5	100	4	0.77	356	93.0	44,500	2.0
entry 10	Ni5	1000	4	0.90	417	93.7	46,500	1.8

^a^ Polymerization conditions: butadiene, 2 mL, 1.4 g; toluene, total volume 16 mL; Ni, 10 μmol; temperature, 22 °C; ^b^ N = moles of butadiene polymerized per mol of Ni per hour; ^c^ determined by ^1^H NMR, the remaining units are 1,2; and ^d^ determined by GPC.

## Data Availability

The original contributions presented in this study are included in the article/Supplementary Material. Further inquiries can be directed to the corresponding author(s).

## Supplementary Materials

The following supporting information can be downloaded at: https://www.mdpi.com/article/10.3390/molecules30234655/s1. Figures S1–S5: FT-IR spectra of the nickel complexes; Figure S6: vials with the five complexes **Ni1**–**Ni5**; Figure S7: complex **Ni4** in solid state (left) and dichloromethane solution (right); Figure S8: Ni-P bond length in **Ni1** and **Ni3** fall within the range 2.2378(5)–2.2483(9) Å, placing them near the mean value of the distance distribution; Figures S9–S13 and Tables S1–S4: further crystallographic details; Figures S14–S16: ^1^H and ^13^C NMR spectra of the polymers; and Figure S17: FT-IR spectra of the polymers.
