# Supplementary material for: Nickel Phosphine Complexes: Synthesis, Characterization, and Behavior in the Polymerization of 1,3-Butadiene
Source: Molecules. 2025 Dec 4;30(23):4655. doi: 10.3390/molecules30234655 (PMC12693259; doi:10.3390/molecules30234655)
Supplement: Supplementary file 1 [file molecules-30-04655-s001.zip › molecules-3981928-supplementary.pdf]

Supporting Information

Type of the Paper (Article)

Nickel Phosphine Complexes: Synthesis, Characterization and Behavior in the Polymerization of 1,3-Butadiene.

Massimo Guelfi[1,2*], Giulio Bresciani[1], Guido Pampaloni[3], Anna Sommazzi[4], Francesco Masi[5], Benedetta Palucci[6], Simona Losio[6], Giovanni Ricci[6*]

1 Università di Pisa, Dipartimento di Chimica e Chimica Industriale, via Moruzzi 13, I-56124 Pisa, Italy; giulio.bresciani@unipi.it (G.B.)
2 Centro per l'Integrazione della Strumentazione Scientifica dell'Univesità di Pisa (C.I.S.U.P), Università di Pisa, I-56124 Pisa, Italy.
3 Scientific Advisor, via E. Fiumalbi 29, I-56025 Pontedera (PI), Italy; pampaloniguido55@gmail.com (G.P.)
4 Scientific Advisor, viale Giovanni XXIII 34, I-28100 Novara, Italy; anna.sommazzi14@gmail.com (A.S.)
5 Scientific Advisor, via Galvani 7, I-26866 Sant'Angelo Lodigiano (LO), Italy; rolando.masi54@gmail.com (F.M.)
6 CNR – Istituto di Scienze e Tecnologie Chimiche "Giulio Natta" (SCITEC), via A. Corti 12, I-20133 Milano, Italy; simona.losio@scitec.cnr.it (S.L.); benedetta.palucci@scitec.cnr.it (B.P.)

*Correspondence: giovanni.ricci@scitec.cnr.it (G.R.); massimo.guelfi@unipi.it (M.G.)

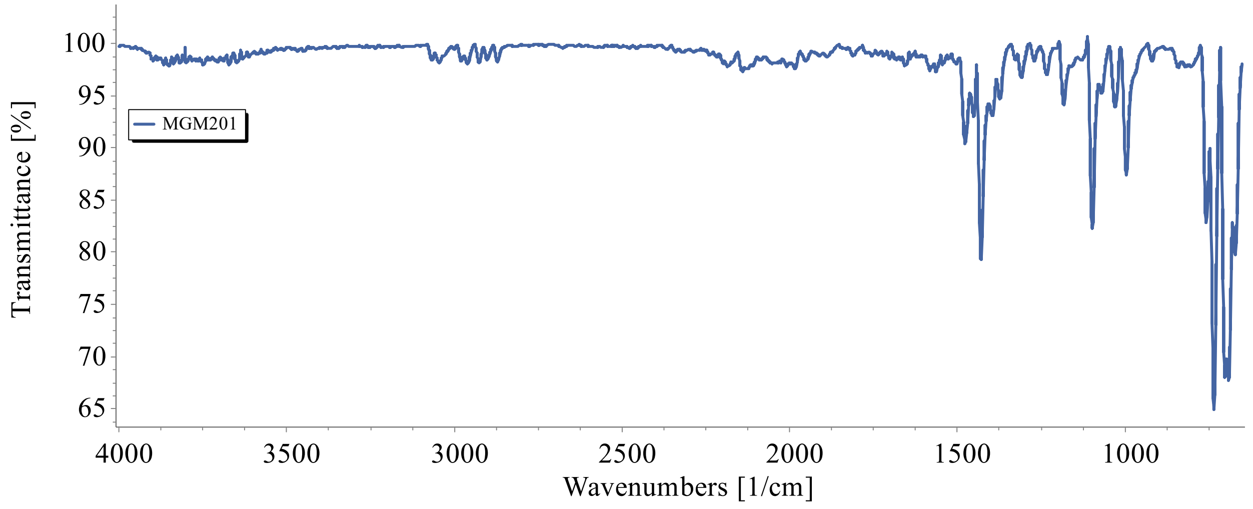

Figure S1. FT-IR spectrum of **Ni1**

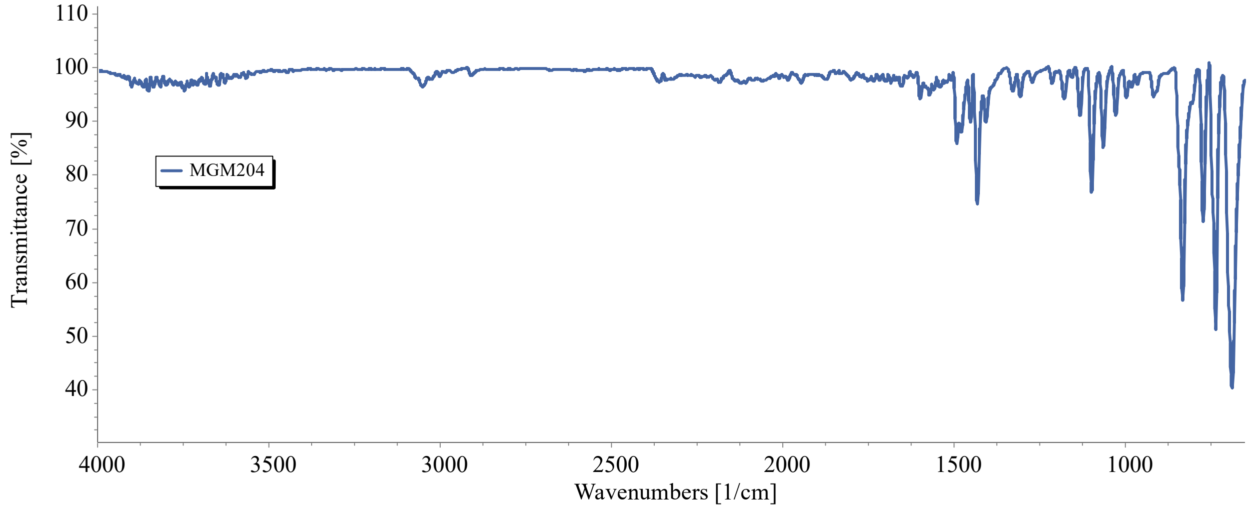

Figure S2. FT-IR spectra of **Ni2**

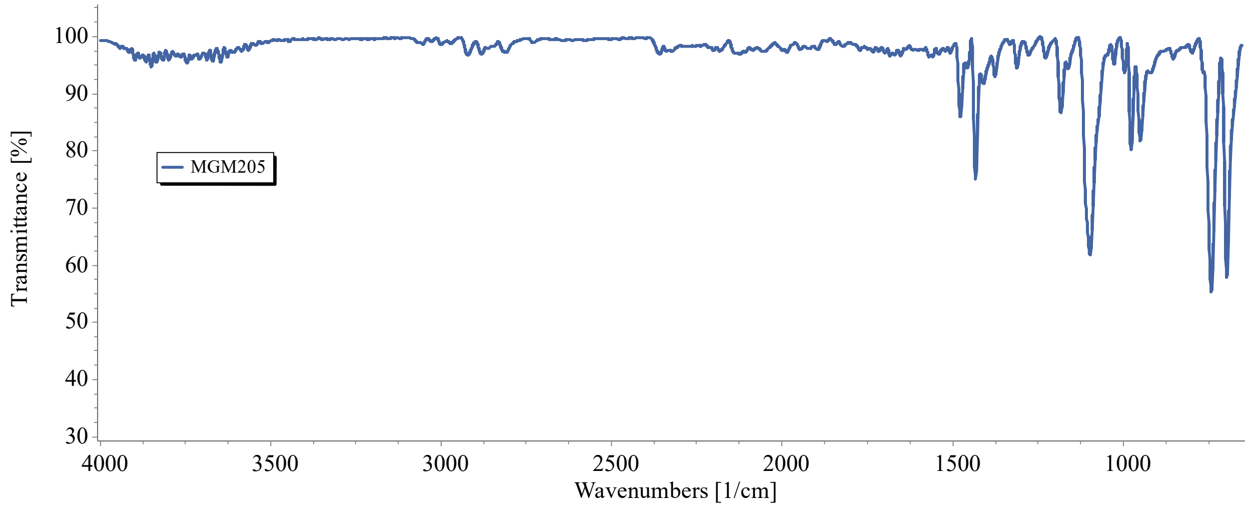

Figure S3. FT-IR spectra of **Ni3**

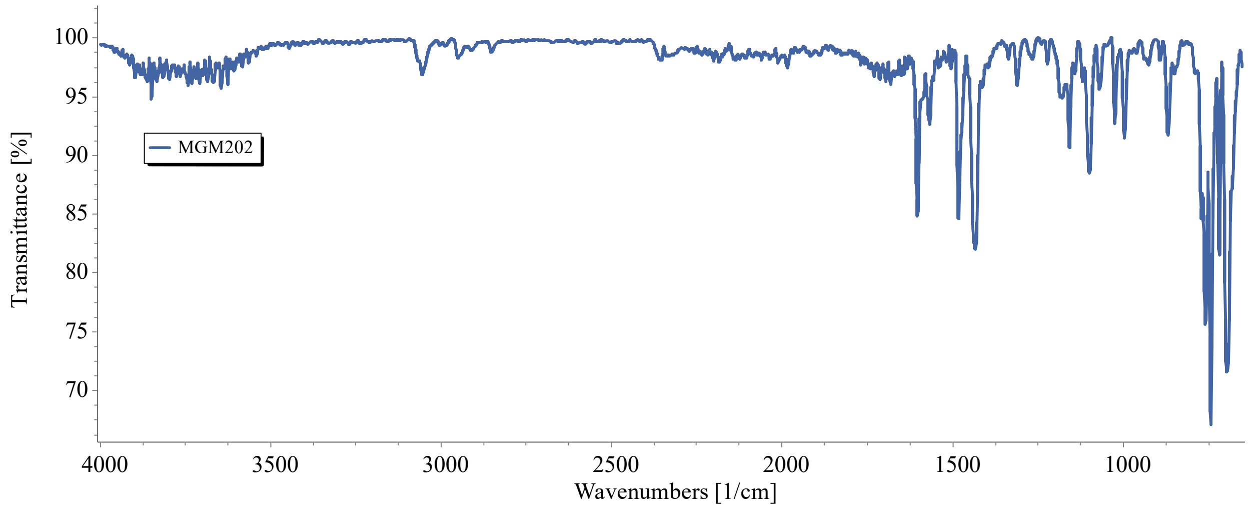

Figure S4. FT-IR spectra of **Ni4**

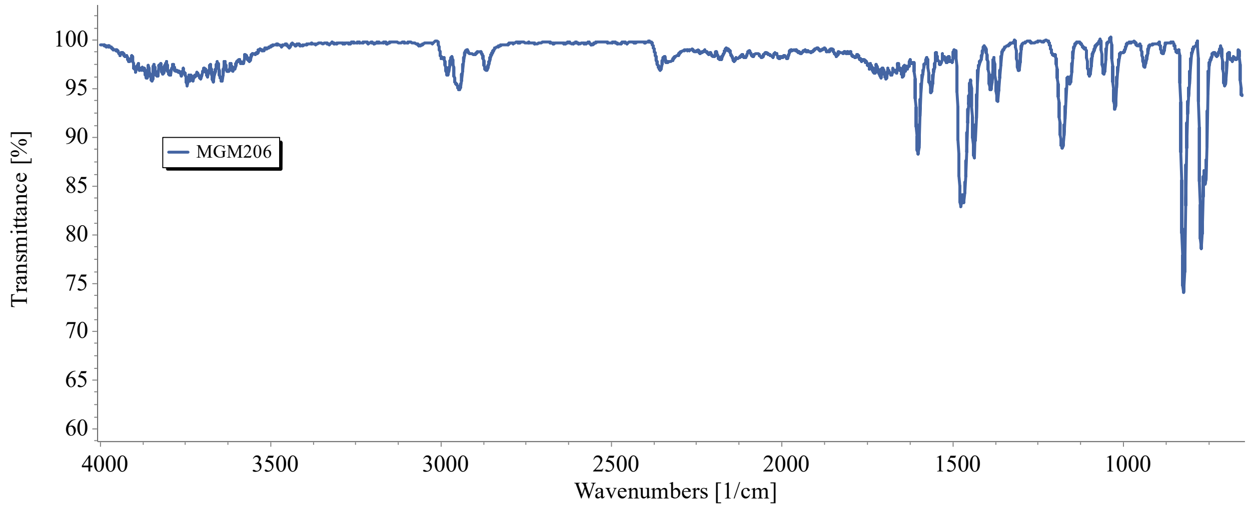

Figure S5. FT-IR spectra of **Ni5**

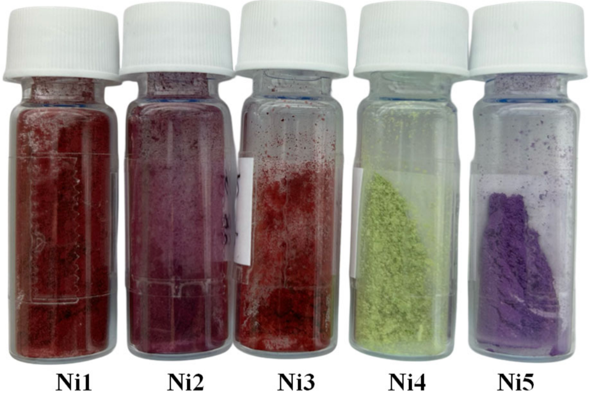

Figure S6. Vials with the five complexes **Ni1-Ni5**

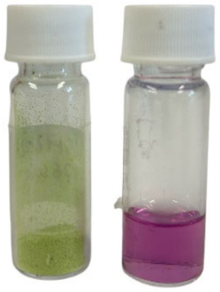

Figure S7. Complex **Ni4** in solid state (left) and dichloromethane solution (right)

Table S1. Bond lengths (Å) and selected bond angles (°) of **Ni1**, **Ni3** and **Ni5** involving Ni, P, Cl and N atoms.

Ni1		Ni3		Ni5	
Ni1-Cl1	2.1536(6)	Ni1-Cl1	2.1491(10)	Ni-N1	2.0005(13)
Ni1-Cl1#1	2.1536(6)	Ni1-Cl1#1	2.1491(10)	Ni-Cl1	2.2159(5)
Ni1-P2	2.2378(5)	Ni1-P1	2.2483(9)	Ni-Cl2	2.2322(5)
Ni1-P2#1	2.2378(5)	Ni1-P2#1	2.2483(9)	Ni-P2	2.3084(4)
Cl1-Ni-P2	90.52(2)	Cl1-Ni-P1	91.30(4)	N1-Ni-Cl1	109.28(4)
Cl1-Ni-P2#1	89.48(2)	Cl1-Ni-P1#1	88.71(4)	N1-Ni-Cl2	102.55(4)
Cl1-Ni-Cl1#1	180.00(4)	Cl1-Ni-Cl1#1	180.00(4)	Cl1-Ni-Cl2	119.09(2)
P2-Ni-P2#1	180.00	P1-Ni-P1#1	180.00	N1-Ni-P2	85.46(4)
				Cl1-Ni-P2	120.306(19)
				Cl2-Ni-P2	112.539(8)

Symmetry transformations used to generate equivalent atoms: #1 -x+1, -y+1, -z+1

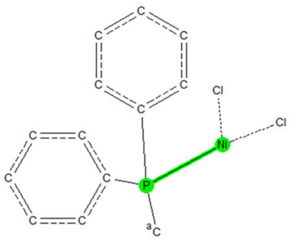

Name	Count	Selected	Mean	Variance	Std. Dev	Mean. Dev	Minimum	Median	Upper quartile	Maximum	Low outliers	High outliers	Total outliers	Lower quartile
DIST1	40	0	2.227	0.004	0.066	0.046	2,115	2.236	2.249	2,401	0	0	0	2.209

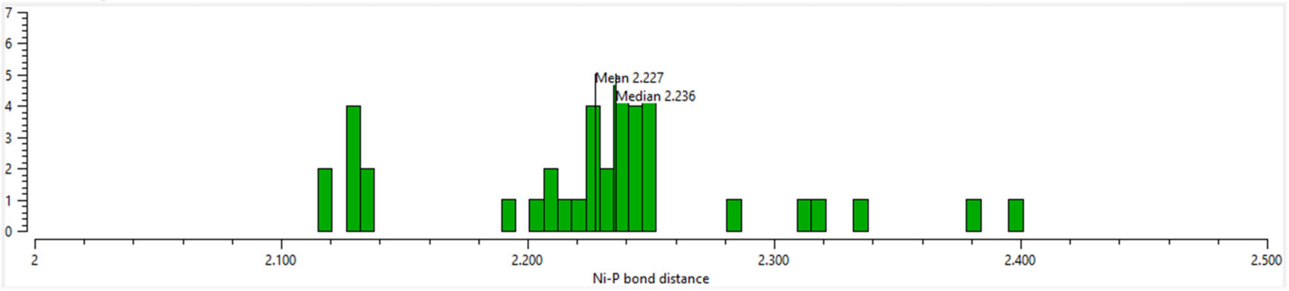

Figure S8. Ni-P bond length in **Ni1** and **Ni3** fall within the range 2.2378(5)-2.2483(9) Å, placing them near the mean value of the distance distribution. Bond length analysis was carried out through a Cambridge Structural Database (CSD) search using ConQuest (2025.1.1), and results were analyzed with Mercury (2025.1.1). The search targeted Cl-Ni-P(Ph)2(R) fragments under the following constraints: the nickel center must exhibit two coordinated chlorine atoms, a phosphorus atom bonded to two phenyl groups and to a generic alkyl group.

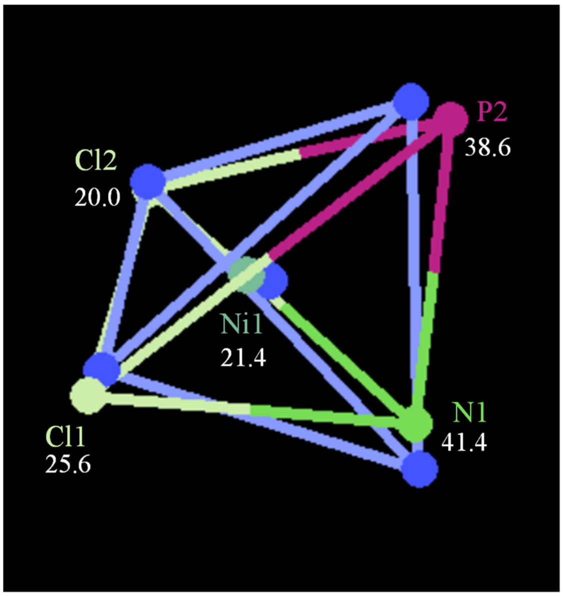

Figure S9. Representation of the distortion of the coordination polyhedron (green: nitrogen atom; violet: phosphorous atom; light green: chlorine atoms; blue green: nickel atom) with respect to the ideal tetrahedron (light blue) for complex **Ni5**. The polyhedron is oriented to highlight the maximum deviation from the Platonic solid (numerical values in pm): N1 (41.4), P2 (38.6), Cl1 (25.6), Cl2 (20.0) and Ni1 (21.4).

Table S2. Hydrogen bonds for **Ni1**, **Ni3** and **Ni5** [Å and °].

Complex	D-H…□	d(H…□)	d(D…□)	<(DH□)
Ni1	C5-H5…Cl1	3.014	3.638	125.82
	C10-H10…Cl1	2.958	3.698	137.53
Ni3	C13-H13B…O1□	2.544	3.438	153.12
	C5-H5…Cl1	2.958	3.597	127.08
Ni5	C6-H6□…Cl2	2.726	3.662	162.34
	C8-H8B□…Cl2	2.913	3.837	161.78

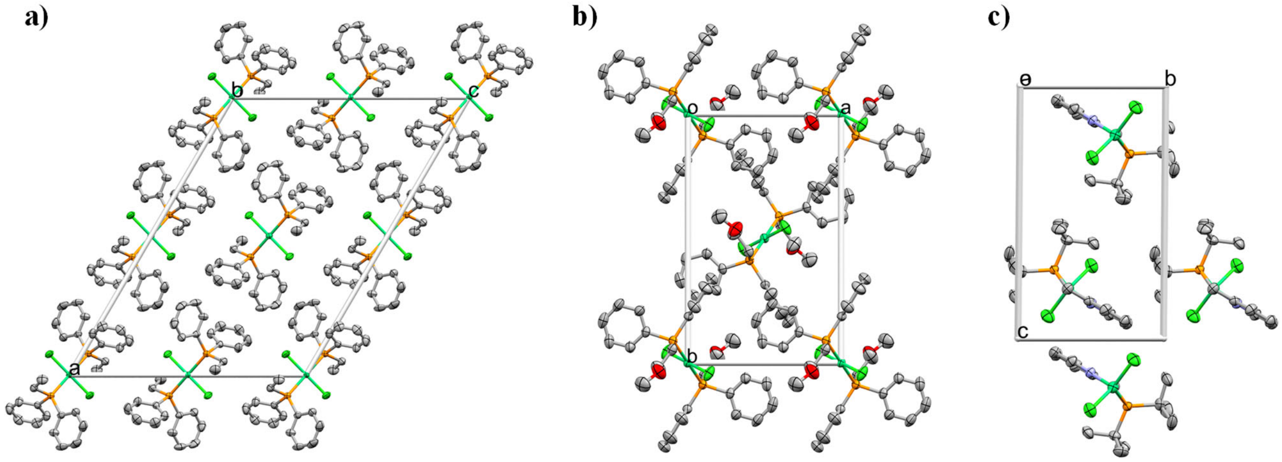

Figure S10. Representation of the unit cells of the three complexes. a) **Ni1** viewed along the *b*-axis; b) **Ni3** viewed along the *c*-axis; c) **Ni5** viewed along *a*-axis. Hydrogen atoms have been omitted for clarity

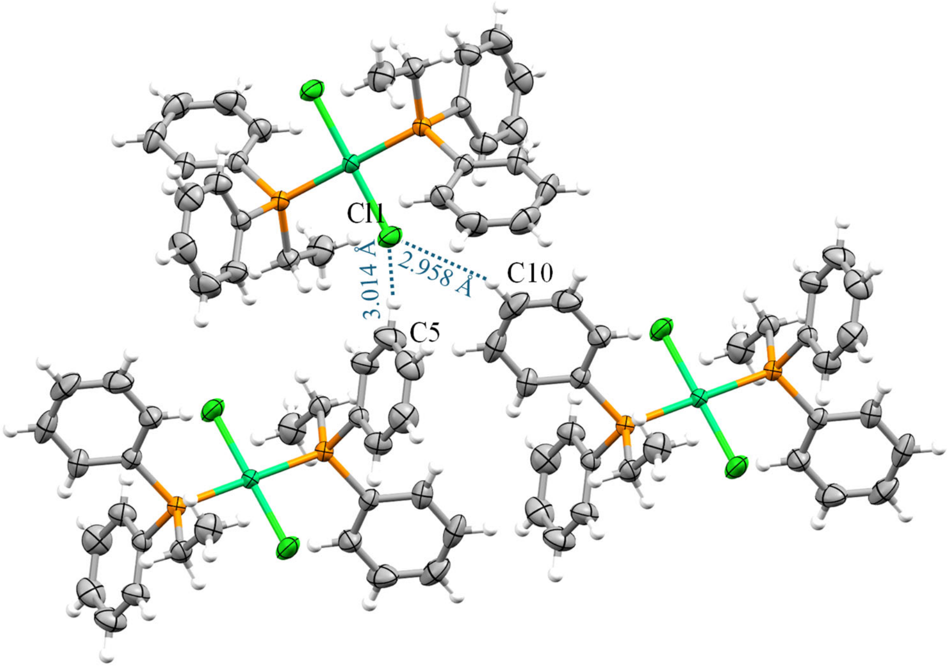

Figure S11. Contacts in **Ni1**

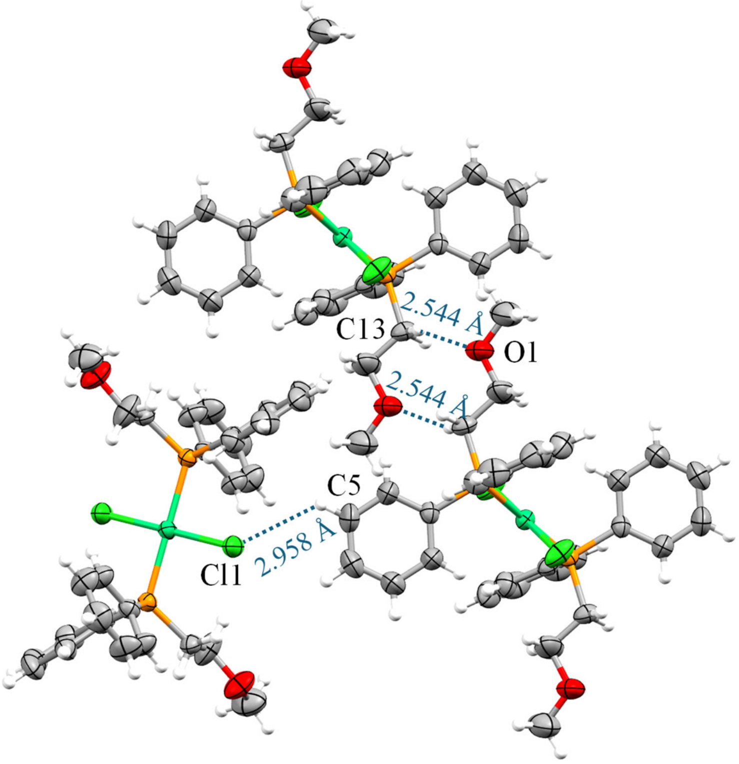

Figure S12. Contacts in **Ni3**

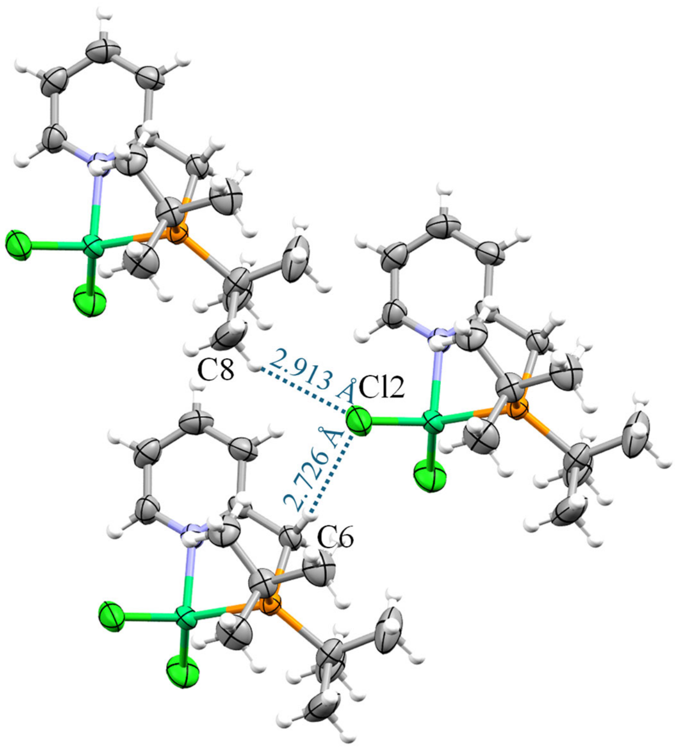

Figure S13. Contacts in **Ni5**

Table S3. Total $\%V_{bur}$ and $\%V_b$ values, that is the volume buried in each quadrant of the space for **Ni1**, **Ni3** and **Ni5** complexes.

Complex	**Ni1**	**Ni3**	**Ni5**
$\%V_{bur}$	64.7	64.0	52.0
Quadrant $\%V_b$			
SW	62.3	61.4	66.1
NW	67.2	66.6	53.8
NE	62.3	61.4	42.4
SE	67.2	66.6	45.8

Polymerization

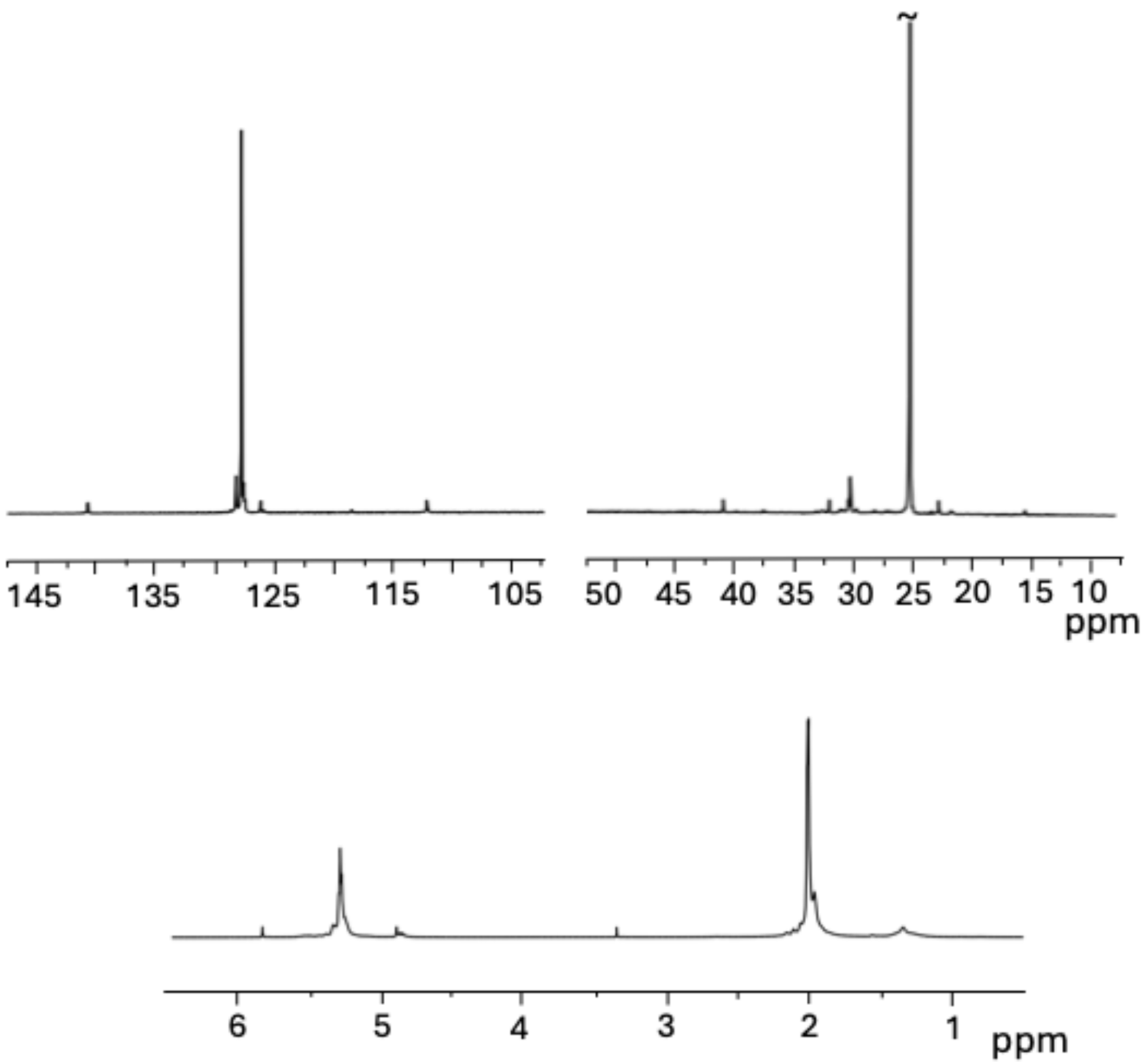

Figure S14. ^{13}C (up) and ^{1}H (down) NMR of polybutadiene of Table 1, entry 1.

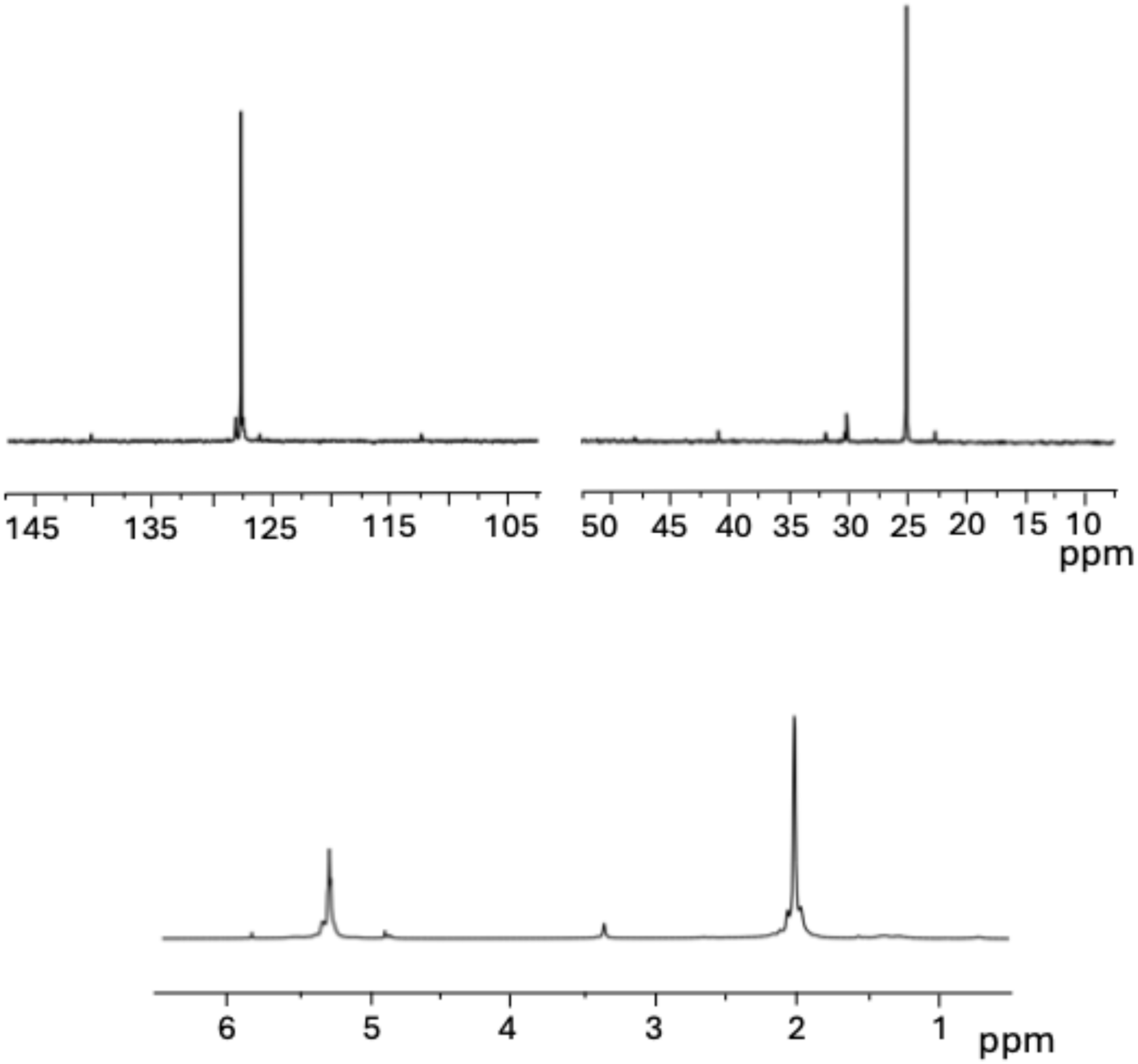

Figure S15. ^{13}C (up) and ^{1}H (down) NMR of polybutadiene of Table 1, entry 4.

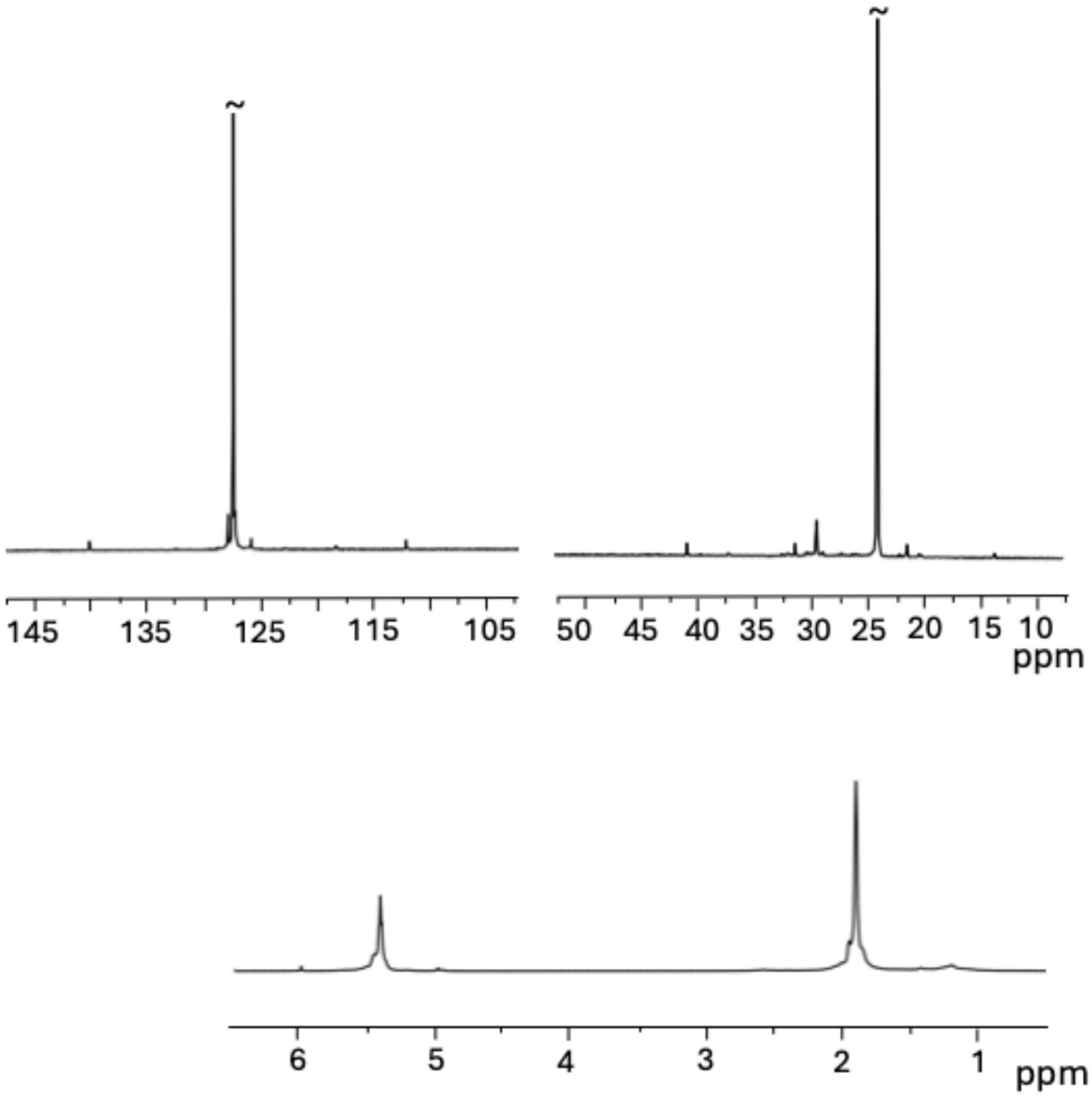

Figure S16. ^{13}C (up) and ^{1}H (down) NMR of polybutadiene of Table 1, entry 6.

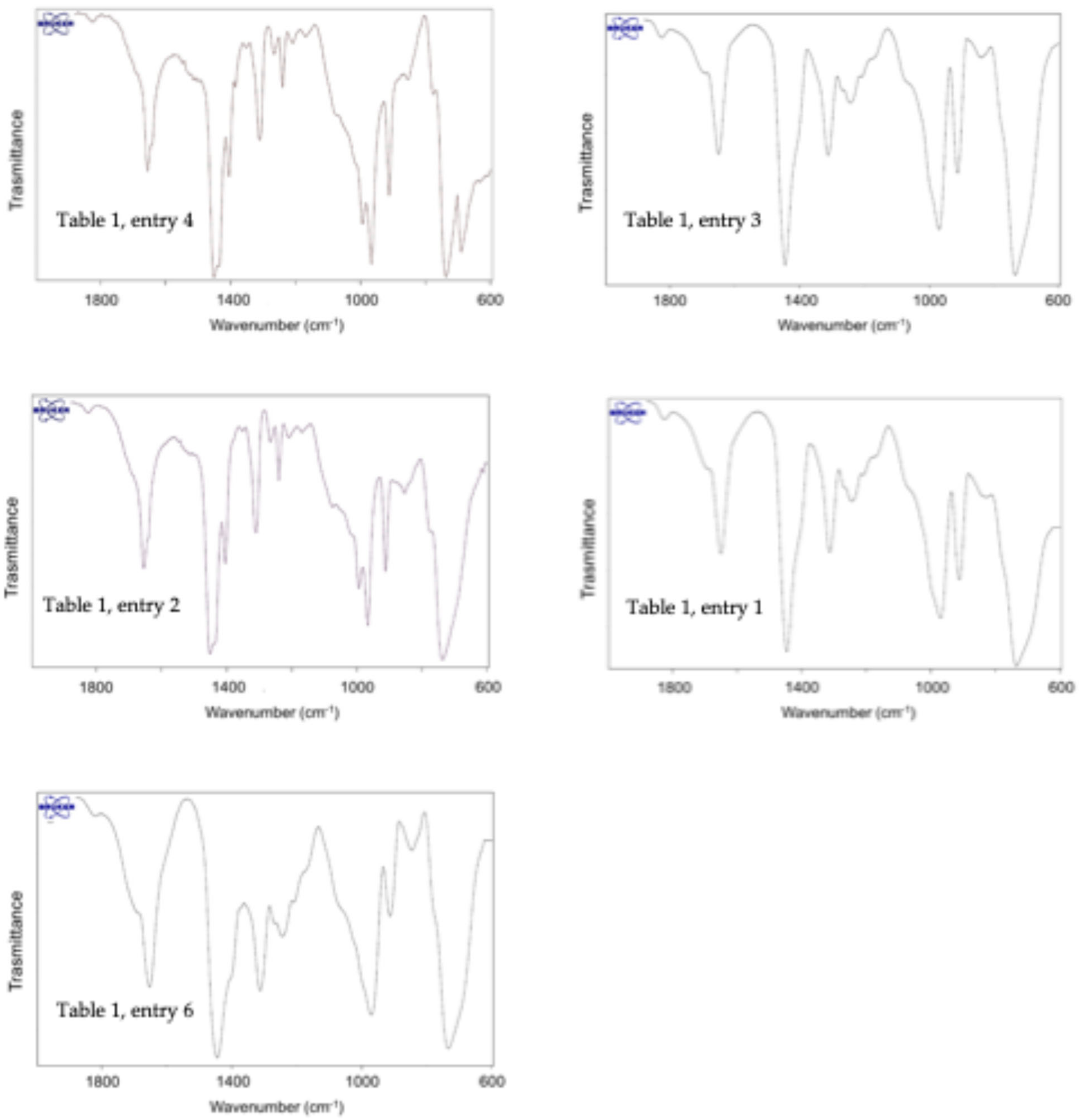

Figure S17. FT-IR spectra of polybutadienes of Table 1.

Table S4. Sample, crystal data, data collection and structure refinement for **Ni1**, **Ni3** and **Ni5.**

CCDC ID			
Compound	Ni1	Ni3	Ni5
Formula	$C_{28}H_{30}Cl_2NiP_2$	$C_{30}H_{36}Cl_2NiO_2P_2$	$C_{14}H_{24}Cl_2NNiP$
FW, g mol^{-1}	558.07	620.14	366.92
T, K	301(2)	301(2)	300(2)
λ, Å	0.71073	1.54178	0.71073
Crystal system	Monoclinic	Monoclinic	Triclinic
Space group	*C*2/c	$P2_1$/n	*P*-1
a, Å	23.575(3)	9.678(4)	7.4818(2)
b, Å	7.6114(12)	15.698(7)	9.0950(3)
c, Å	17.305(2)	10.079(4)	14.7868(4)
α, °	90	90	85.5700(10)
β, °	120.332(4)	94.78(4)	77.7720(10)
γ, °	90	90	66.3410(10)
Cell volume, Å^3	2680.1(7)	1525.9(11)	900.68(5)
Z	4	2	2
Density calculated, g cm^{-3}	1.383	1.350	1.353
□bsorption coefficient, mm^{-1}	1.058	3.713	1.450
F(000)	1160	648	384
Crystal size, mm	0.026x0.065x0.080	0.027x0.031x0.069	0.086x0.125x0.170
θ limits, °	2.00 to 25.06	5.23 to 66.62	2.44 to 27.50
Reflection collected	25563	21100	40727
Independent reflection	2384	2671	4129
Data/restraints/parameters	2384/0/152	2671/0/171	4129/0/189
Goodness of fit on F^2	1.048	1.125	1.046

R_1 ($I > 2\sigma(I)$)	0.0270	0.0684	0.0263
wR_2 ($I > 2\sigma(I)$)	0.0642	0.1873	0.0689
R_1 (all data)	0.0333	0.1015	0.0297
wR_2 (all data)	0.0680	0.2270	0.0709
Largest diff. peak and hole, e Å^{-3}	0.333 and -0.249	1.576 and -0.994	0.436 and -0.278
